# Identification of Minimal *p53* Promoter Region Regulated by MALAT1 in Human Lung Adenocarcinoma Cells

**DOI:** 10.3389/fgene.2017.00208

**Published:** 2018-03-26

**Authors:** Keiko Tano, Rena Onoguchi-Mizutani, Fouzia Yeasmin, Fumiaki Uchiumi, Yutaka Suzuki, Tetsushi Yada, Nobuyoshi Akimitsu

**Affiliations:** ^1^Isotope Science Center, The University of Tokyo, Tokyo, Japan; ^2^Department of Gene Regulation, Faculty of Pharmaceutical Sciences, Tokyo University of Science, Noda-shi, Chiba-ken, Japan; ^3^Department of Computational Biology, Graduate School of Frontier Sciences, The University of Tokyo, Tokyo, Japan; ^4^Department of Bioscience and Bioinformatics, Kyushu Institute of Technology, Kitakyushu, Japan

**Keywords:** noncoding RNA, TP53, transcription, genetic, adenocarcinoma, promoter regions, genetic

## Abstract

The MALAT1 long noncoding RNA is strongly linked to cancer progression. Here we report a MALAT1 function in repressing the promoter of *p53 (TP53)* tumor suppressor gene. *p21* and *FAS*, well-known p53 targets, were upregulated by MALAT1 knockdown in A549 human lung adenocarcinoma cells. We found that these upregulations were mediated by transcriptional activation of p53 through MALAT1 depletion. In addition, we identified a minimal MALAT1-responsive region in the P1 promoter of *p53* gene. Flow cytometry analysis revealed that MALAT1-depleted cells exhibited G1 cell cycle arrest. These results suggest that MALAT1 affects the expression of p53 target genes through repressing *p53* promoter activity, leading to influence the cell cycle progression.

## Introduction

Recent large-scale transcriptome analyses have revealed that transcription is spread throughout the mammalian genome (>90%), including in noncoding regions. This yields large numbers of noncoding RNAs (ncRNAs) (Birney et al., 2007), including small ncRNAs (20–200 nt), and long ncRNAs (lncRNA) (>200 nt). Increasing numbers of studies have revealed the unique features and functions of lncRNAs in broad biological processes as well as development of diseases (Mercer et al., 2009). Considering their critical roles in cellular and molecular mechanisms, we cannot exclude the involvement of lncRNAs from molecular analyses of biological processes and disease pathogenesis.

Recent studies have reported several lncRNAs exhibiting cancer-specific expression patterns (Tano and Akimitsu, 2012). Metastasis associated lung adenocarcinoma transcript 1 (MALAT1) is a well-known cancer-related lncRNA over 8,000 nt in length. MALAT1 was originally identified in early-stage nonsmall cell lung cancer (NSCLC) with a high propensity for metastasis (Ji et al., 2003). Although Malat1 is not essential in living mice maintained under normal breeding conditions (Nakagawa et al., 2012; Zhang et al., 2012), MALAT1 has an important role in the study of human cancer: MALAT1 is overexpressed in many solid tumors (Lin et al., 2007; Gutschner et al., 2013a). In addition, high expression levels of MALAT1 correlated with poor prognosis in patients with NSCLC (Ji et al., 2003). MALAT1 contributes in metastasis phenotype of human lung cancer cells (Tano et al., 2010; Gutschner et al., 2013b). These results highlight that revealing the function of MALAT1 is important to understand the cancer biology.

MALAT1 functions in regulating gene expression in several capacities. Intracellularly, MALAT1 is stably retained in the nucleus (Miyagawa et al., 2010; Tani et al., 2012). MALAT1 specifically localizes to nuclear speckles, which are subnuclear structures enriched for pre-mRNA splicing factors (Hutchinson et al., 2007), and regulates alternative splicing by modulating the distribution and levels of active pre-mRNA splicing factors (SR proteins) in nuclear speckles (Tripathi et al., 2010). Another report demonstrated MALAT1 involvement in regulated transcriptional programs (Yang et al., 2011). MALAT1 was shown to regulate the relocation of growth control genes from the repressive environment of polycomb bodies (PcGs) to the gene activation milieu of interchromatin granules (ICGs) in response to growth signals by interacting with unmethylated Pc2. This leads to the promotion of E2F1 sumoylation and activation of transcription of genes associated with growth control. In addition, identification of genomic region interacted with MALAT1 revealed that MALAT1 associates with transcriptionally active genes (Engreitz et al., 2014; West et al., 2014). These results highlight the distinct role of MALAT1 in the regulation of gene expression program.

We previously demonstrated a link between characteristics of cancer metastasis and gene regulation by MALAT1 (Tano et al., 2010). We showed that MALAT1 promotes cell migration, which is one of the most important features for metastasis, through regulation of several migration-related genes at the transcriptional and/or post-transcriptional level (Tano et al., 2010). We also performed microarray analysis of MALAT1-knockdown cells and found that MALAT1 was involved in the repression of several genes associated with tumor suppression.

In this study, we re-examined the previous microarray data and found that several MALAT1-regulated genes were *p53 (TP53)* target genes, such as *p21 (CDKN1A)* and *FAS*, suggesting the possibility that MALAT1 is involved in the repression of these genes through p53. We showed that upregulation of both *p21* and *FAS* in MALAT1- depleted A549 lung adenocarcinoma cells was repressed by knockdown of *p53* and inhibition of p53 activity by PFT-α. Further, we found that depletion of MALAT1 leads to upregulation of p53 through activation of *p53* promoter. We identified −153 to −111 of the P1 *p53* promoter as a MALAT1-responsive region. This is the first report showing that MALAT1 affects expression of p53 target genes through negative regulation of specific elements in the *p53* promoter. Finally, we showed that depletion of MALAT1 resulted in cell cycle arrest in G1. Together our results indicate that MALAT1 may have additional functions in repressing tumor suppression to promote cancer progression.

## Materials and methods

### Cell culture and RNA interference

A549 and H1299 (kindly supplied by Dr. Hideki Matsumoto, Fukui University, Japan) cells were grown at 37°C with 5% CO_2_ in Dulbecco's modified Eagle's medium (DMEM) or RPMI 1640 medium, respectively, supplemented with 10% fetal bovine serum and penicillin/streptomycin.

RNA interference was performed using Lipofectamine RNAiMAX (Invitrogen, Tokyo, Japan), according to the manufacturer's instructions. The siRNA sequences were as follows (sense/antisense): *p53* (siRNA 1), 5′-GTGAGCGCTTCGAGATGTTCC-3′/5′-AACATCTCGAAGCGCTCACGC-3′; and *p53* (siRNA 2), 5′-gacTccagTggTaaTcTacTT-3′/5′- gTagaTTaccacTggagTcTT-3′. The MALAT1 siRNA and the negative control siRNA sequences were described previously (Tano et al., 2010). Efficient reduction of each gene by siRNA was confirmed by quantitative real-time PCR analysis.

### Plasmid constructs

The human *p53* promoter pGL2 (Basic) luciferase plasmids containing *p53* promoter fragments (356, 200, and 100 bp) were purchased from Addgene (MA, USA). For the construction of 5′-end deletion mutant reporter plasmids, each fragment was amplified by PCR and cloned into the pGL2 basic reporter vector. The primers used for PCR cloning were as follows: pGL2-177bp-F-SacI, 5′-cagaccGAGCTCctcctccccaactccatttc-3′; pGL2-165bp-F-SacI, 5′-cagaccGAGCTCtccatttcctttgcttcctc-3′; pGL2-148bp-F-SacI, 5′-cagaccGAGCTCctccggcaggcggattac-3′; pGL2-140bp-F-SacI, 5′-cagaccGAGCTCggcggattacttgcccttac-3′; pGL2-130bp-F-SacI, 5′-cagaccGAGCTCttgcccttacttgtcatggcg-3′; pGL2-122bp-F-SacI, 5′-cagaccGAGCTCacttgtcatggcgactgtcc-3′; pGL2-110bp-F-SacI, 5′-cagaccGAGCTCgactgtccagctttgtgccag-3′; and pGL2-Re-HindIII, 5′-aatcccAAGCTTctagacttttgagaagctcaaaacttttag-3′. The pGL2-200bp *p53* promoter plasmid was used as a template.

For the construction of deletion mutant plasmids, in which a part of the MALAT1 response element was deleted, we performed site-directed mutagenesis using primers as follows: p53pro-del1-F, 5′-GCTTCCTCCGGCAGGCGG-3′; p53pro-del1-Re, 5′-AAATGGAGTTGGGGAGGAGGGTGC-3′; p53pro-del2-F, 5′-GGATTACTTGCCCTTACTTGTCATG-3′; p53pro-del2-Re, 5′-CCGGAGGAAGCAAAGGAAATG-3′; p53pro-del3-F, 5′-CCTTACTTGTCATGGCGACTG-3′; and p53pro-del3-Re, 5′-TAATCCGCCTGCCGGAGG-3′.

### Reverse transcription and quantitative real-time PCR analysis

Total RNA was prepared using the RNAiso Plus kit (Takara Bio, Shiga, Japan), and 500 ng of RNA was reverse transcribed to produce cDNA with the PrimeScript RT Master Mix (Takara Bio). Real-time PCR was carried out with the Thermal Cycler Dice using the SYBR Premix Ex Taq II (Takara Bio, Shiga, Japan). The sequences of primer sets used in this analysis were shown in Table 1.

**Table 1:** Primers for p53 target genes.

**Gene**	**Forward**	**Reverse**
p21	tcactgtcttgtacccttgtgc	ggcgtttggagtggtagaaa
FAS	gtggacccgctcagtacg	tctagcaacagacgtaagaacca
DNAJC15,	agattcagatagagtgccagcat	tggaatgaacccaagatttgt
p53	cgtgtggagtatttggatgac	ttgtagtggatggtggtacagtc
pre-mRNA of p53	gtggaaggaaatttgcgtgt	accacccttaacccctcct
BTG2	gcgagcagaggcttaaggt	gggaaaccagtggtgtttgta
SULF2	aagcacggctccgactact	gtgcggaagaagctcacg
HMOX1	gggtgatagaagaggccaaga	agctcctgcaactcctcaaa
RPS27L	tctatcccggaagttgatgc	caagctcagccctaccagac
ACTA2	ccctgaagtacccgatagaaca	ggcaacacgaagctcattg
FDXR	gtgacacagccgtgattctg	cttcgtgatgtccgttctctc
GAPDH	gcaccgtcaaggctgagaac	tggtgaagacgccagtgga

### RT-PCR

Total RNA was extracted using RNAiso Plus (Takara Bio), and 500 ng of RNA was reverse transcribed to produce cDNA with the PrimeScript RT Master Mix (Takara Bio). PCR was performed on 1/10 (2 μl) of the cDNA using Ex Taq HS polymerase (Takara Bio). PCR conditions were 30 cycles of 98°C for 10 s, 55°C for 30 s and 72°C for 1 min. PCR products were separated on 2% agarose gels.

### Western blot analysis

Cells were lysed with RIPA buffer containing 125 mM Tris-HCl (pH 8.0), 375 mM sodium chloride, 2.5 mM EDTA, 0.25% sodium dodecyl sulfate, 2.5% Triton X-100, 0.25% sodium deoxycholate, 1 mM PMSF, 5 μg/ml Leupeptin, and 1 μl Aprotinin Solution (Wako, Osaka, Japan), and centrifuged at 15,000 rpm to remove debris. Protein extracts (10 μg) were resolved by sodium dodecyl sulfate 10% polyacrylamide gel electrophoresis and transferred to a PVDF membrane. After blocking with 3% BSA in TBST, the blot was probed with the DO-1 monoclonal anti-human p53 antibody (Medical and Biological Laboratories, Aichi, Japan), followed by peroxidase labeled anti-mouse antibody (no. NIF 825, GE Healthcare). For α-tubulin detection, rabbit anti-α-tubulin antibody (Medical and Biological Laboratories) and peroxidase labeled anti-rabbit antibody (no. NA934VS; GE Healthcare) were used. The horseradish peroxidase-labeled antibodies were detected by Immobilon Western Chemiluminescent HRP Substrate (Millipore) using a Lumino image analyzer, LAS4000 (Fujifilm).

### Massive transcriptional start site (TSS) analysis

TSS analysis was performed as described in Tani et al. (2012). Briefly, cells transfected with control or MALAT1 siRNA were harvested and RNA was extracted using RNAiso Plus. Thirty micrograms of the obtained total RNA was subjected to oligo-capping by treatment of BAP, TAP, and RNA oligo ligation. After DNase I treatment, polyA-containing RNA was selected by oligo-dT powder. First strand cDNA was synthesized from random hexamers and amplified with 15 cycles of PCR using Gene Amp PCR kits (Perkin Elmer). The PCR fragments were size fractionated by 12% polyacrylamide gel electrophoresis and the 150–250 bp fraction was recovered. The quality and quantity of the obtained single-stranded first strand cDNAs were assessed by BioAnalyzer (Agilent).

One nanogram of the size-fractionated cDNA was used for the massively paralleled sequencing by an Illumina GA Sequencer. Clusters (15,000–20,000) were generated per tile and 36 cycles of the sequencing reactions were performed according to the manufacturer's instructions. The 36-base long tags corresponding to the 5′-ends of transcripts were generated by the sequencer. The obtained sequences were mapped onto human genomic sequences (hg18 of UCSC Genome Browser) using the sequence alignment program Eland.

### Luciferase assays

Luciferase assays were performed using the dual-luciferase reporter assay system (Promega). Cells were co-transfected with the *p53* promoter reporter plasmids containing firefly luciferase and internal control reporter plasmids containing Renilla luciferase using Lipofectamine 2000 (Invitrogen), according to the manufacturer's instructions. At 24 h after transfection, cells were harvested and luciferase activity was measured following the manufacturer's protocol.

## Results and discussion

### Upregulation of both p21 and FAS in MALAT1-knockdown A549 cells was mediated by p53

**Figure 1:**
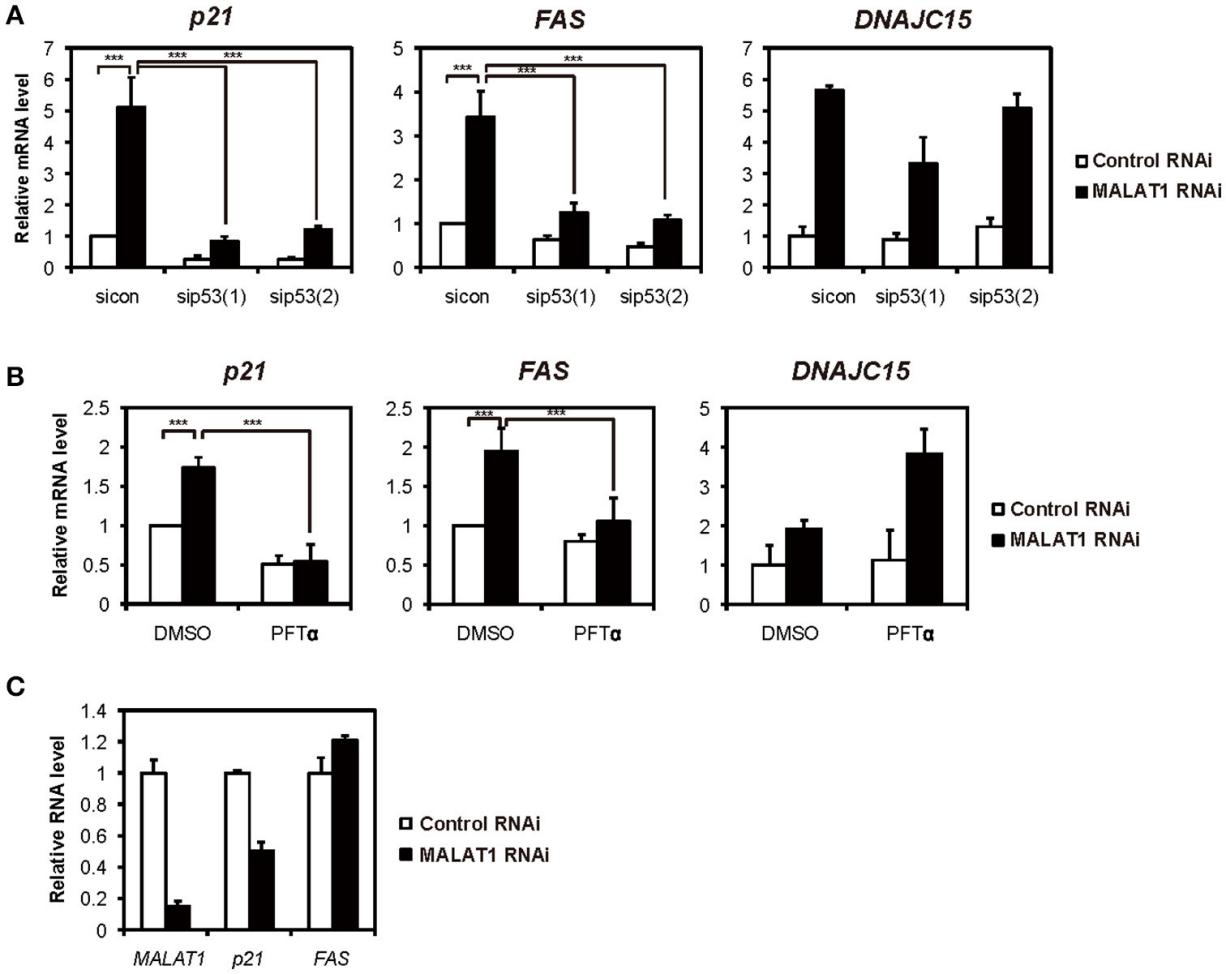
Increased expression levels of both *p21* and *FAS* mediated by p53 in MALAT1-knockdown cells. **(A,B)** Real-time PCR analyses were performed to assess the effects of knockdown of *p53* by p53 siRNA 1 (sip53(1)) and p53 siRNA 2 (sip53(2)) **(A)** or inhibition of p53 by PFT-α in the presence of doxorubicin **(B)** on the expression of *p21, FAS* and *DNAJC15* genes in MALAT1-knockdown cells. Relative mRNA expression levels are presented as ratios to the level of that in control cells. Data are presented as means ± standard deviation (*SD*) of three independent experiments (^***^P < 0.001, two-way ANOVA followed by Bonferroni *t*-test as post-hoc test), except for *DNAJC15*, which shows the average of duplicate experiments. **(C)** p53-null H1299 cells were transfected with MALAT1 siRNA, and *p21* and *FAS* expression levels were analyzed by real-time PCR. Values represent the means ± *SD* of duplicate measurements.

Previously, we showed that several p53 target genes, including *p21* and *FAS*, were upregulated by knockdown of MALAT1 in A549 cells, in which *p53* is intact (Tano et al., 2010). This prompted the hypothesis that MALAT1 represses the expression of *p21* and *FAS* genes through p53 activity. To test this hypothesis, we examined whether the p53 target genes were upregulated through p53 activity in MALAT1-knockdown cells. First, we confirmed the upregulation of p53 target genes upon MALAT1 knockdown (Figure 1A and Figure S3). We then found that upregulation of *p21* and *FAS* in MALAT1-knockdown cells was repressed by siRNA-mediated *p53* depletion (Figure 1A). In contrast, upregulation of *DNAJC15*, which is not a p53 target gene, was not repressed by *p53* depletion in MALAT1-knockdown cells. Generally, knockdown efficiency is not 100%; therefore, we still detected some upregulation of *p21* and *FAS* mRNAs upon MALAT1 knockdown even in the p53-knockdown cells. To further investigate whether p53 is involved in the upregulation of *p21* and *FAS* mRNAs, we examined Pifithrin-α (PFTα), a specific inhibitor of p53, on the upregulation of *p21* and *FAS* in MALAT1-knockdown cells. The increased expression levels of *p21* and *FAS* in MALAT1-knockdown cells were inhibited by PFT-α, and this inhibitory effect was not observed with *DNAJC15* (Figure 1B). Furthermore, we found that MALAT1-knockdown-mediated upregulation of both *p21* and *FAS* was not observed in p53-null H1299 cells (Figure 1C). These results suggest that upregulation of *p21* and *FAS* in MALAT1-knockdown cells was mediated by p53 activity.

**Figure 2:**
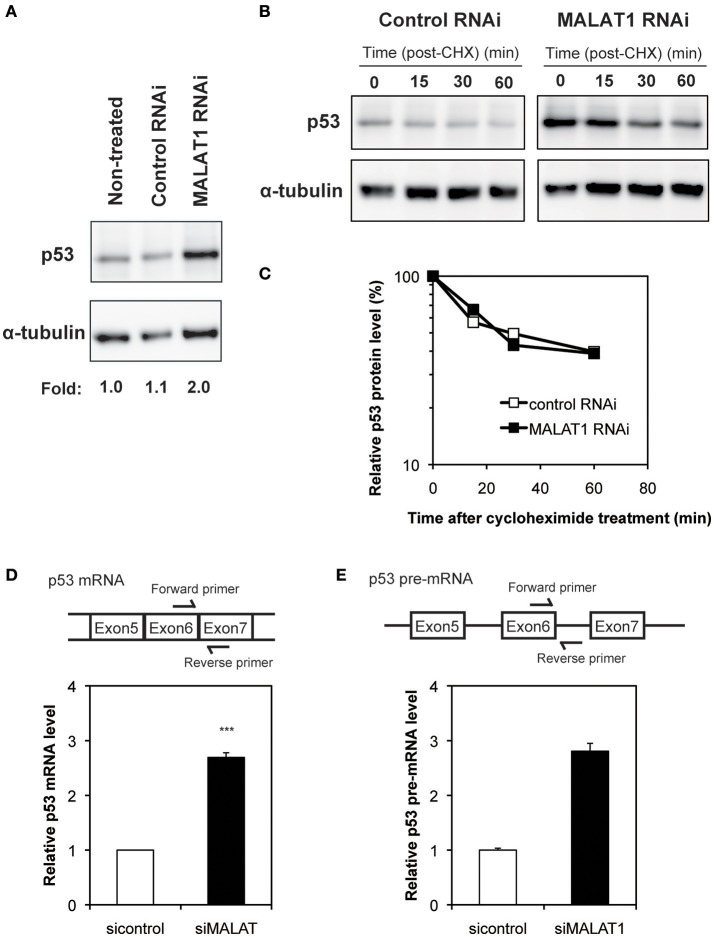
Increased expression level of p53 in MALAT1-knockdown cells. **(A)** Expression levels of p53 and α-tubulin were determined by Western blotting. **(B)** Determination of p53 protein stability in MALAT1-knockdown cells. Cells were treated with cycloheximide (CHX) (100 μM) to inhibit further protein synthesis and harvested at the indicated times. Remained Proteins of p53 and α-tubulin were measured by western blot analysis. The signal intensities of p53 bands were normalized by those of α-tubulin bands. **(C)** Quantitative analysis of p53 protein levels in **(B)** using a lumino image analyzer. **(D)** Expression levels of *p53* mRNA were analyzed by real-time RT-PCR. Data are presented as means ± *SD* of three independent experiments (^***^P < 0.001, Student's *t*-test). A schematic diagram to detect matured *p53* mRNAs is shown at upper area. **(E)** Pre-mRNA level of *p53* was examined by real-time RT-PCR. Values represent the means ± *SD* of duplicate measurements. A schematic diagram to detect pre-matured *p53* mRNAs is shown at upper area.

To investigate the mechanism by which MALAT1 affects p53 activity, we next examined the expression levels of p53 in MALAT1-knockdown cells. Western blot analysis revealed that the p53 protein levels in MALAT1-knockdown cells were increased (Figure 2A). Since changes in p53 protein levels have been attributed to increases in p53 protein stability, we investigated the half-life of p53 protein in MALAT1-knockdown cells. The half-life of p53 in MALAT1-knockdown cells was not changed compared with control cells (Figures 2B,C), suggesting that the increase in p53 protein in MALAT1-knockdown cells was not due to increased protein stability.

We then determined whether changes in *p53* mRNA levels were the basis of increased p53 protein in MALAT1-knockdown cells. qRT-PCR revealed that the level of *p53* mRNA was increased by approximately three-fold in MALAT1-knockdown cells (Figure 2D). Pre-mRNA level of *p53* gene was also upregulated in MALAT1-knockdown cells (Figure 2E). We also observed upregulation of mature and pre-mature *p53* mRNAs upon MALAT1 depletion in HCT116 cell, a human colorectal carcinoma cell line expressing normal p53 (Figure S4). These results suggest that knockdown of MALAT1 resulted in increased p53 expression by increasing *p53* mRNA levels.

### MALAT1 negatively regulates p53 promoter activity

**Figure 3:**
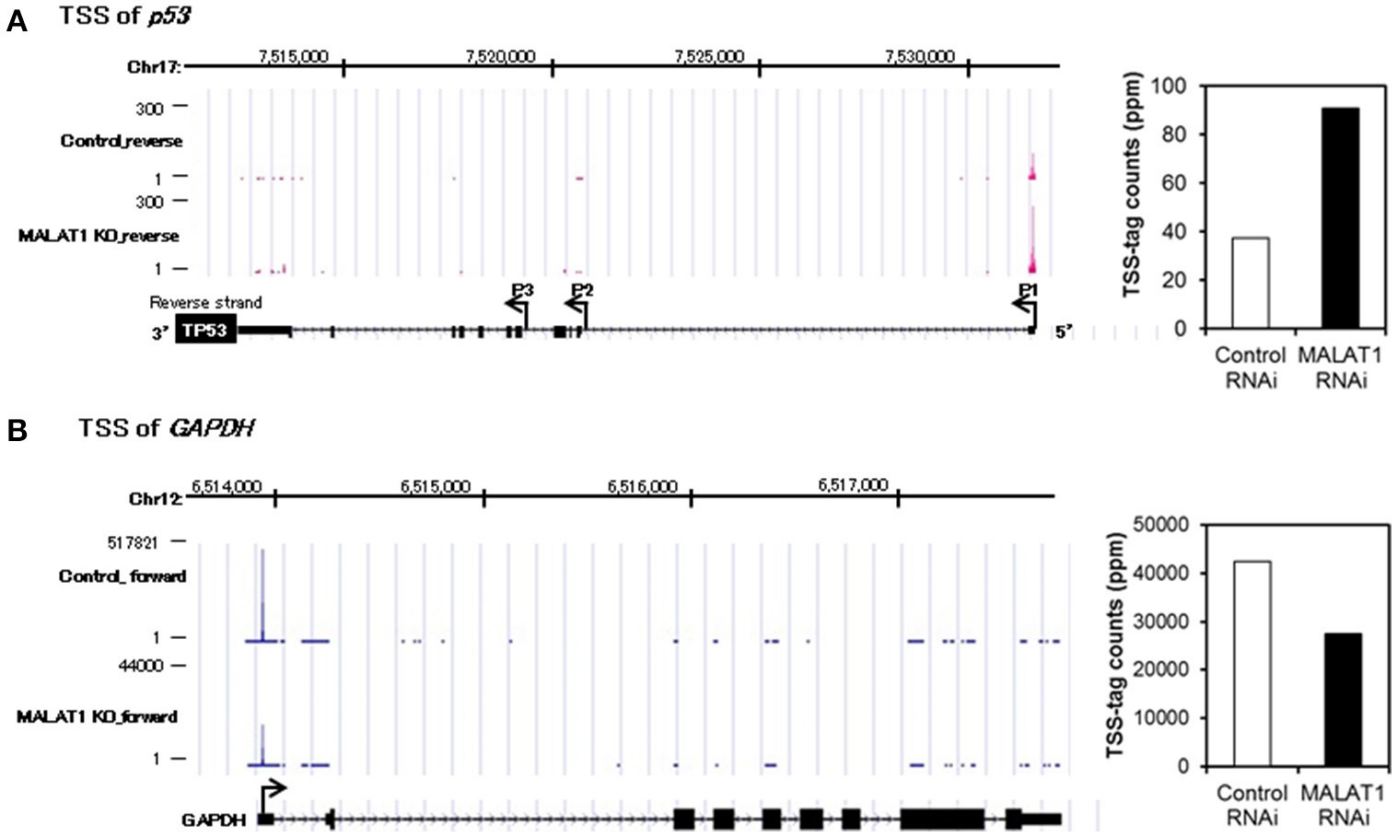
Transcriptional start site (TSS) analysis in MALAT1-knockdown cells. **(A,B)** TSS-tags counts mapped at the corresponding genomic regions in *p53* **(A)** or *GAPDH* **(B)**. The bar graph on the right indicates TSS-tag counts at the position of *p53* P1 promoter **(A)** or *GAPDH* promoter. TSS-tags belonging to the corresponding TSS clusters were counted. TSS-tag counts were divided by the total number of mapped TSS-tags to calculate TSS-tag ppm (parts per million).

To further investigate the mechanism for increased expression of *p53* mRNA in MALAT1-knockdown cells, we analyzed *p53* promoter activity. Since the *p53* gene (*TP53*) has several alternative promoters (p53 P1 promoter, which is located within a 356 bp region upstream of the major transcription start site of the *p53* gene, p53 P2 promoter and p53 P3 promoter) (Tuck and Crawford, 1989; Hollstein and Hainaut, 2010), we first performed massive transcriptional start site (TSS) analysis (Suzuki et al., 1997; Tsuchihara et al., 2009), which can easily monitor the genome-wide positions of TSSs. The number of TSS-tags corresponds to the number of transcripts that initiate from the site, therefore TSS analysis enables the determination of regions that can upregulate transcription of the *p53* gene in MALAT1-knockdown cells. TSS analysis revealed that the number of TSS-tags at the position of the P1 promoter of *p53* gene was upregulated by more than two-fold in MALAT-1 knockdown cells (Figure 3). In contrast, the TSS-tag counts of *GAPDH* were not increased in MALAT1-knockdown cells. This result suggests that transcription from the p53 P1 promoter was upregulated in MALAT1-knockdown cells. The p53 P1 promoter is located within the noncoding exon 1, and this promoter region can drive the transcript that encodes the active form of p53 protein (Wang and El-Deiry, 2006).

**Figure 4:**
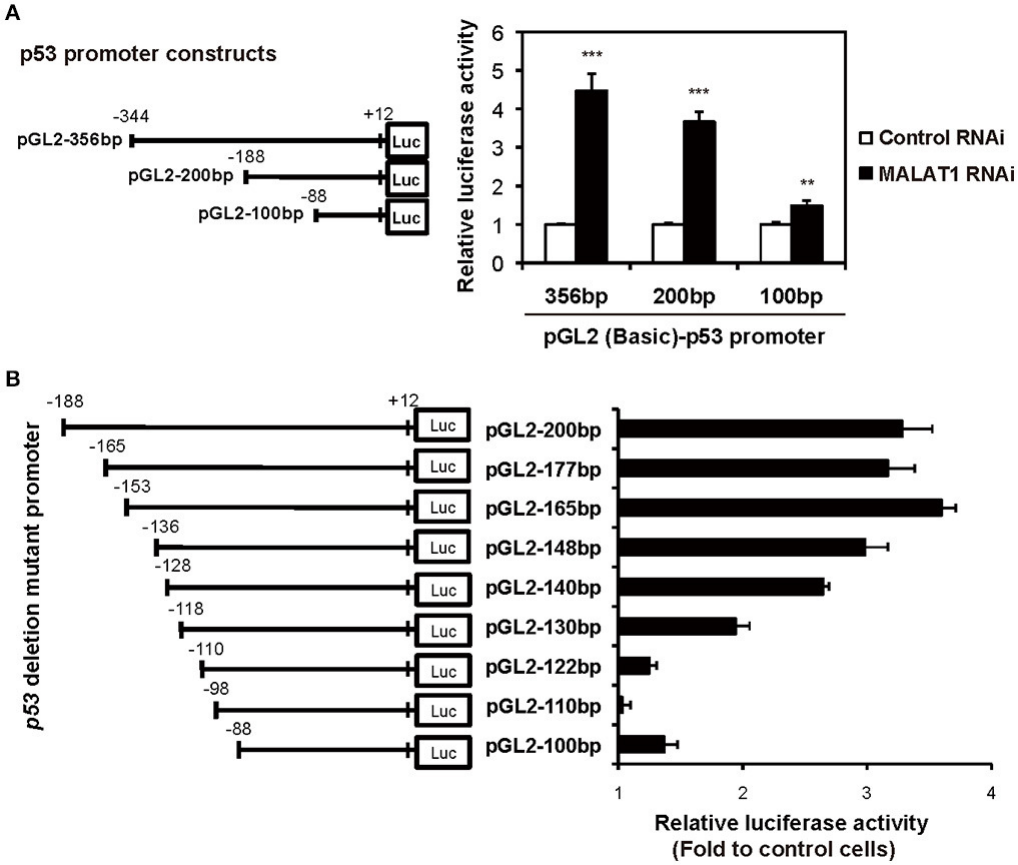
Determination of the MALAT1-responsive region in the human *p53* promoter. **(A)** The activities of the human *p53* promoter regions (pGL2-356,−200, and −100 bp) in MALAT1-knockdown cells were examined using luciferase assays. The data are presented as fold induction compared with the activity in control cells. The *p53* promoter-reporter firefly luciferase activity is indicated relative to the activity of the Renilla luciferase control. All transfections were normalized with equal amounts of pGL2-Control vector. Data are means ± *SD* (*n* = 3 each, ^**^P < 0.01, ^***^P < 0.001, Student's *t*-test). **(B)** The relative luciferase activities of the 5′ deletion mutant promoters in MALAT1-knockdown cells compared with control cells were determined. Data is shown as fold induction compared with the activity in the control cells (activity in the control cells was set at 1.0).

To examine whether the P1 promoter of *p53* gene is activated in MALAT1-knockdown cells, we performed luciferase reporter assays. We transfected MALAT1-knockdown cells with a reporter plasmid containing the P1 promoter, a 356-bp region located −344 to +12 relative to the major TSS (Tuck and Crawford, 1989; Hollstein and Hainaut, 2010). The results showed that promoter activity of the 356 bp region was increased in MALAT1-knockdown cells by more than four-fold compared with control cells, suggesting increased P1 promoter activity in MALAT1-knockdown cells (Figure 4A). The promoter activity of the 200-bp region (−188 to +12) was also elevated in MALAT1-knockdown cells. However, further deletion to 100 bp (−88 to +12) resulted in a two- to three-fold reduction in promoter activity. These results suggest that the P1 promoter region from −188 to −88 contain *cis* elements that may be regulated by MALAT1.

To further elucidate the MALAT1-responsive region in the P1 promoter, we constructed a series of seven 5′ deletion mutant reporter plasmids (pGL2-177,−165,−148,−140,−130,−122, and −110 bp), in which the 5′ ends of the 200-bp region of the *p53* promoter was deleted. The deletion mutant plasmids were transfected into MALAT1-knockdown cells and assayed for luciferase activity. The reporter activity of the *p53* promoter in MALAT1-knockdown cells was gradually reduced by deletion from 165 bp (−153 to +12) to 122 bp (−110 to +12) of the *p53* promoter (Figure 4B). This finding suggests a possible MALAT1-responsive region in the *p53* promoter. We speculated that MALAT1 could regulate the activity of the transcription factors that bind to this region (between −153 to −110) in the *p53* promoter.

**Figure 5:**
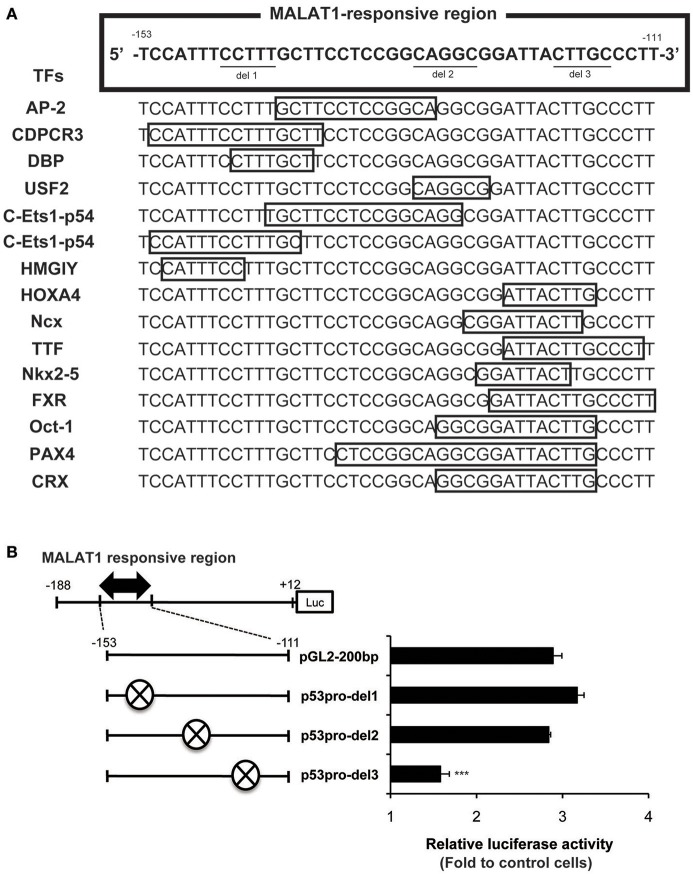
Analysis of the MALAT1-responsive region in the human p53 promoter. **(A)** TRANSFAC database analysis of the MALAT1-response region (−153 to −111) in the *p53* promoter. Transcription factors (TFs, shown on the left) and their binding sites (open boxes) predicted by TRANSFAC are shown. **(B)** The relative luciferase activities of the deletion mutant promoters (p53pro-del1, del2, and del3), in which parts of MALAT1-response elements were deleted, were determined. The deleted sequences (crosshatch, ×) of each mutant promoter are CCTTT (p53pro-del1), CAGGC (p53pro-del2), and CTTGC (p53pro-del3). Data are means ± *SD* (*n* = 3 each, ^***^P < 0.001 compared with the luciferase activity of pGL2-200 bp).

We next evaluated possible binding sites for transcription factors in the MALAT1-responsive region in the *p53* promoter. As summarized in Figure 5A, 15 binding sites for transcription factors were predicted using TRANSFAC database analysis of the MALAT1-response region (−153 to −111). Interestingly, approximately half of these predicted binding sites were located in the 3′-downstream portion of the MALAT1-responsive region. To evaluate the contribution of these binding sites in the 3′-downstream portion of the MALAT1-responsive region, we constructed mutant reporter plasmids with deletions in this region and examined luciferase activity in MALAT1-knockdown cells. A five-nucleotide deletion in the 3′-downstream region (p53pro-del3) resulted in decreased luciferase activity compared with the original sequence in MALAT1-knockdown cells. Other mutants with deletions in other sites, but not in the 3′-downstream element (p53pro-del1 and p53pro-del2), did not show reduced luciferase activity (Figure 5B). These results indicate that binding sites in the 3′-downstream portion of the MALAT1-responsive region is responsible for transcriptional regulation of *p53* by MALAT1. Therefore, we speculated that MALAT1 modulates *p53* promoter activity through regulation of transcription factors predicted to bind to this region.

A previous report suggested that MALAT1 interacts with SR splicing factors, such as SRSF1, which is also known as SF2/ASF, and localizes to nuclear speckles to regulate alternative splicing of pre-mRNA (Tripathi et al., 2010). To rule out the possibility that upregulation of *p53* expression through activation of *p53* promoter is arbitrarily attributed to abnormal alternative splicing caused by knockdown of MALAT1, we examined the effect of depletion of SRSF1 on *p53* expression. Knockdown of *SRSF1* increased the inclusion of exon 19 of *MGEA6*, which is regulated by SRSF1 as well as MALAT1 (Tripathi et al., 2010), indicating changes in alternative splicing in *SRSF1* knockdown cells (Figure S1A). However, knockdown of *SRSF1* did not affect expression of *p53* mRNA (Figure S1B). This result suggests that increased expression of *p53* mRNA in MALAT1-knockdown cells is independent of MALAT1 regulation of alternative splicing. This finding strongly supports our idea that MALAT1 affects *p53* expression through regulating *p53* promoter activity.

MALAT1 is known to localize to nuclear speckles. To determine whether nuclear speckle localization of MALAT1 is necessary for regulation of p53 expression, we examined *p53* expression levels when localization of MALAT1 was disrupted by knockdown of RNPS1 or SRm160, which are nuclear speckle proteins that contribute to MALAT1 nuclear speckle localization (Miyagawa et al., 2010). Expression level of *p53* was not increased by disrupting the localization of MALAT1 to nuclear speckles using knockdown of RNPS1 and SRm160 genes instead of MALAT1 knockdown (Figure S2). We confirmed that knockdown of RNPS1 and SRm160 had little effect on MALAT1 expression (less than two-fold). This result indicates that localization of MALAT1 to nuclear speckles is not necessary for controlling expression of *p53*. This finding indicates the possibility that MALAT1 functions in mechanisms at other nuclear structures in addition to nuclear speckles.

### Depletion of MALAT1 induces G1 cell cycle arrest

**Figure 6:**
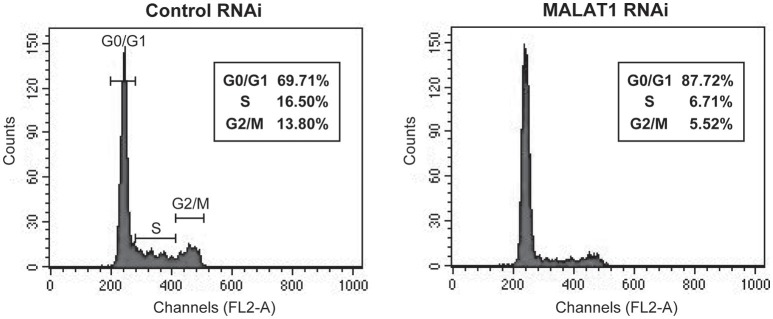
MALAT1-knockdown cells display cell cycle arrest in G1 phase. Cells transfected with control or MALAT1 siRNA were harvested, stained with propidium iodide, and analyzed for DNA content with FACScalibur.

*p53* mRNA levels are tightly regulated during the cell cycle, with its transcription induced before DNA synthesis and a peak production at the G1-S cell cycle transition (Wang and El-Deiry, 2006). In addition, p21, which is upregulated by p53 in MALAT1-knockdown cells, is a major mediator of G1 cell cycle arrest (Chen et al., 1996). To explore the biological significance of MALAT1-mediated p53 expression, we examined whether MALAT1 knockdown leads to cell cycle arrest in G1. Flow cytometry analysis revealed that depletion of MALAT1 in A549 cells exhibited a higher proportion of cells in G0/G1 (approximately 90%) and lower proportion in S (7%) and G2/M (6%), compared with control cells (Figure 6), indicating that depletion of MALAT1 leads to cell cycle arrest in G1 phase. Therefore, the function of MALAT1 in regulating *p53* promoter activity would contribute to the regulation of cell cycle progression, especially in G1 phase.

In the present study, we demonstrated that upregulation of *p21* and *FAS* in MALAT1-depleted cells was mediated by p53. We also showed that depletion of MALAT1 increased the expression of p53 at both the protein and mRNA levels. In addition, we found that depletion of MALAT1 led to increased activity of the *p53* promoter through specifically affecting 3′-downstream elements in the MALAT1-responsive region in *p53*. Together results suggest that MALAT1 reduces the expression of *p21* and *FAS* through repression of *p53* promoter activity.

We also demonstrated that depletion of MALAT1 in A549 cells resulted in G1 cell cycle arrest. Tripathi et al. showed that depletion of MALAT1 in human diploid fibroblasts leads to defects in cell cycle progression in G1/S and activation of p53 (Tripathi et al., 2013). However, the mechanisms by which MALAT1 regulates p53 expression were not determined. Herein, we propose a novel function for MALAT1 in regulating *p53* promoter activity and controlling cell cycle progression.

Our data identified eight transcription factors that were predicted to bind to 3′-downstream portion of the MALAT1-responsive region, including HOXA4, Ncx, TTF, Nkx2-5, FXR, Oct-1, PAX4, and CRX. Although p53 is a well-known tumor suppressor subject to multiple regulations at the transcriptional level (Tripathi et al., 2013), none of these candidate transcription factors were previously identified as *p53* regulatory factors. Of these, Nkx2-5 and FXR are most the likely candidates of *p53* regulatory factors because TSS analysis revealed that the number of TSS-tags of their target genes, *ECE-1* and *SOCS3*, respectively, were increased in MALAT1-depleted cells. Therefore, these factors may be responsible for the MALAT1-mediated regulation of the *p53* promoter. Further studies are required to elucidate the precise molecular mechanism of *p53* promoter regulation by MALAT1 with responding transcription factors.

Together our findings help elucidate the novel regulation of the *p53* promoter by the long noncoding RNA, MALAT1, and further our understanding of MALAT1 function in cancer biology.

## Author contributions

KT: carried out mot experiments; RO-M, YS, and TY: carried out part of FACS, NGS, and bioinformatics, respectively; FY carried out part of RT-qPCR; KT, FU, and NA: designed this study; KT, RO-M, YS, TY, FU, and NA: wrote the manuscript.

### Conflict of interest statement

The authors declare that the research was conducted in the absence of any commercial or financial relationships that could be construed as a potential conflict of interest. The reviewer AM and handling Editor declared their shared affiliation.

## Supplementary material

Figure S1 Increased expression of *p53* mRNA in MALAT1-knockdown cells is independent of alternative splicing regulated by MALAT1. **(A)** Changes in alternative splicing in *SRSF1* knockdown cells are shown by increased inclusion of exon 19 of *MGEA6*. RT-PCR analysis was performed using primers specific for SRSF1-regulated alternative exons in *MGEA6*. Alternative exon-included (upper band) and exon-excluded bands (lower band) are shown. **(B)** Quantitative real-time PCR analysis of *SRSF1* expression levels (left) and *p53* mRNA levels (right) in *SRSF1* knockdown cells compared with control cells. Values represent the means ± *SD* of duplicate measurements.

Figure S2 Localization of MALAT1 to nuclear speckles is not necessary for regulation of *p53* expression. **(A,B)** Quantitative real-time PCR analysis of *p53* mRNA levels in RNPS1- **(A)** or SRm160- **(B)** knockdown cells. Values represent the means ± *SD* of duplicate measurements. **(C,D)** Quantitative real-time PCR analysis of MALAT1 expression levels in RNPS1- **(C)** or SRm160- **(D)** knockdown cells. Values represent the means ± *SD* of duplicate measurements.

Figure S3 Increased expression levels of indicated p53 target mRNAs upon MALAT1 knockdown. Real-time PCR analyses determined the expression levels of indicated RNAs those are normalized by GAPDH mRNA. Data are presented as means ± errors of two independent experiments.

Figure S4 Increased expression levels of pre-matured and matured *p53* mRNAs in MALAT1-knockdown cells. Real-time PCR analyses were performed to assess the indicated RNAs those are normalized by GAPDH mRNA. Data are presented as means±standard deviation (*SD*) of three independent experiments (^*^*P* < 0.05, ^**^*P* < 0.01, Student's *t*-test).
