# Supplementary figures and images for: Identification of Minimal p53 Promoter Region Regulated by MALAT1 in Human Lung Adenocarcinoma Cells
Source: Front Genet. 2018 Mar 26;8:208. doi: 10.3389/fgene.2017.00208 (PMC5879451; doi:10.3389/fgene.2017.00208)

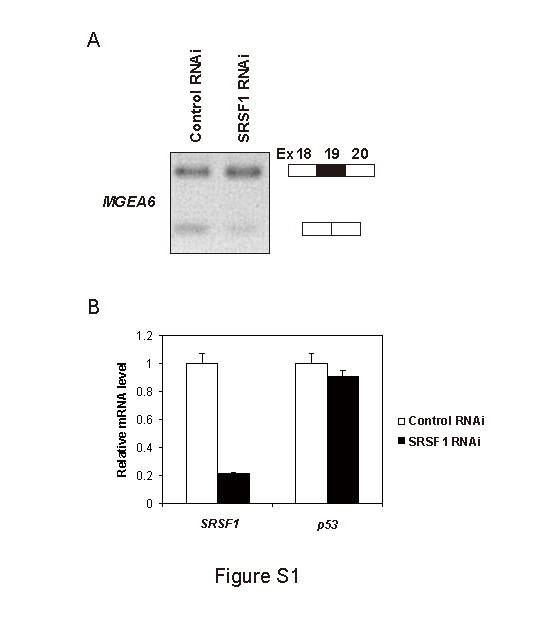

Supplement: Figure S1 — Increased expression of p53 mRNA in MALAT1-knockdown cells is independent of alternative splicing regulated by MALAT1. (A) Changes in alternative splicing in SRSF1 knockdown cells are shown by increased inclusion of exon 19 of MGEA6. RT-PCR analysis was performed using primers specific for SRSF1-regulated alternative exons in MGEA6. Alternative exon-included (upper band) and exon-excluded bands (lower band) are shown. (B) Quantitative real-time PCR analysis of SRSF1 expression levels (left) and p53 mRNA levels (right) in SRSF1 knockdown cells compared with control cells. Values represent the means ± SD of duplicate measurements. [file Image1.tif]

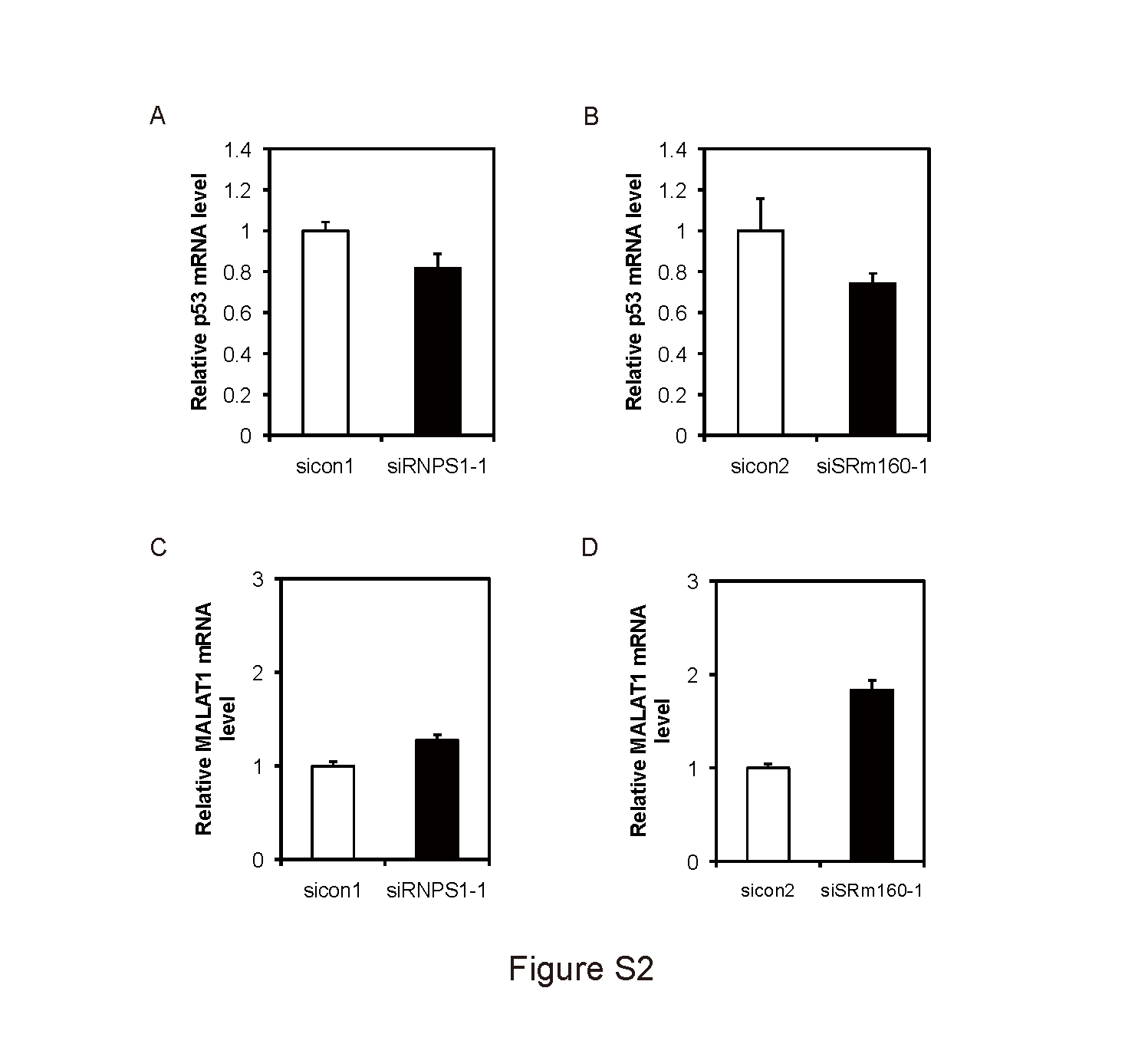

Supplement: Figure S2 — Localization of MALAT1 to nuclear speckles is not necessary for regulation of p53 expression. (A,B) Quantitative real-time PCR analysis of p53 mRNA levels in RNPS1- (A) or SRm160- (B) knockdown cells. Values represent the means ± SD of duplicate measurements. (C,D) Quantitative real-time PCR analysis of MALAT1 expression levels in RNPS1- (C) or SRm160- (D) knockdown cells. Values represent the means ± SD of duplicate measurements. [file Image2.tif]

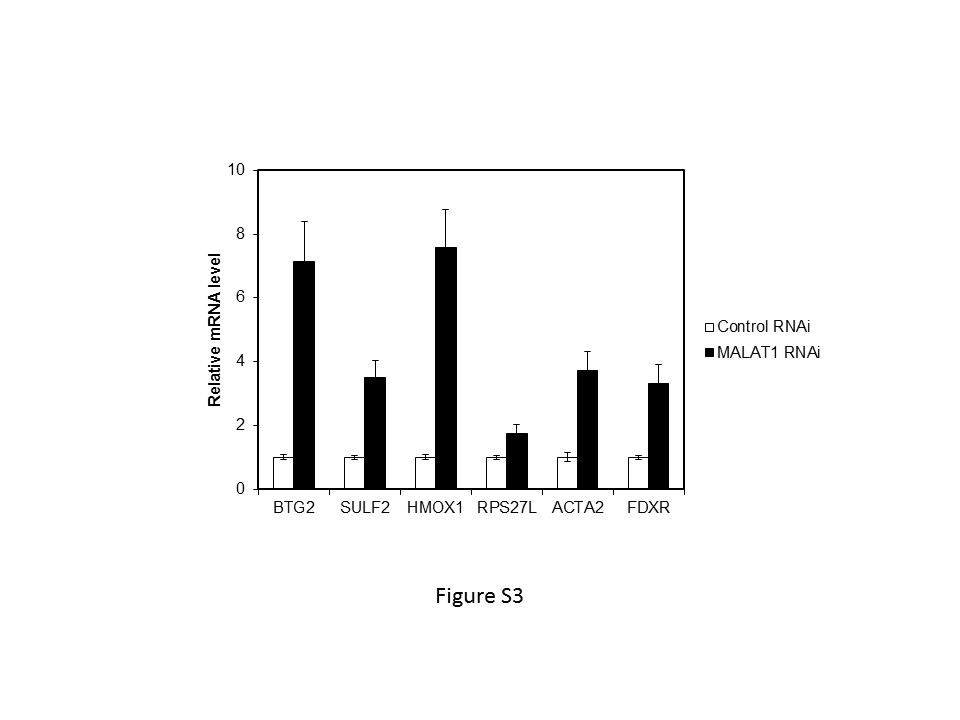

Supplement: Figure S3 — Increased expression levels of indicated p53 target mRNAs upon MALAT1 knockdown. Real-time PCR analyses determined the expression levels of indicated RNAs those are normalized by GAPDH mRNA. Data are presented as means ± errors of two independent experiments. [file Image3.tif]

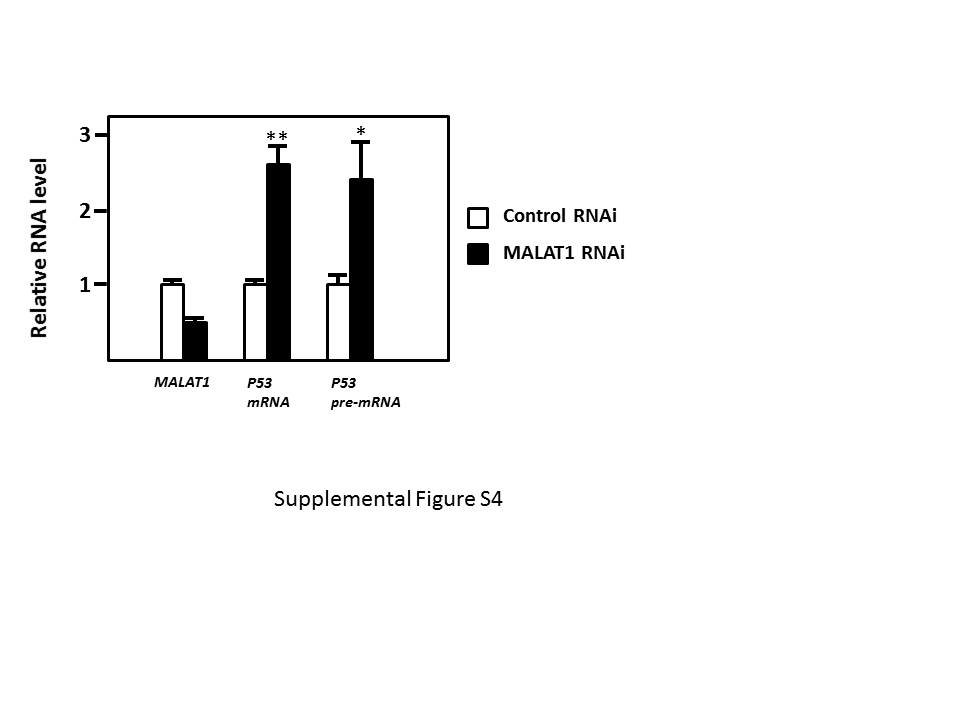

Supplement: Figure S4 — Increased expression levels of pre-matured and matured p53 mRNAs in MALAT1-knockdown cells. Real-time PCR analyses were performed to assess the indicated RNAs those are normalized by GAPDH mRNA. Data are presented as means±standard deviation (SD) of three independent experiments (*P < 0.05, **P < 0.01, Student's t-test). [file Image4.tif]
